# Supplementary material for: NOX4 Deficiency Exacerbates the Impairment of Cystatin C-Dependent Hippocampal Neurogenesis by a Chronic High Fat Diet
Source: Genes (Basel). 2020 May 19;11(5):567. doi: 10.3390/genes11050567 (PMC7291165; doi:10.3390/genes11050567)
Supplement: Supplementary file 1 [file genes-11-00567-s001.pdf]

Supplemental information

NOX4 deficiency exacerbates the impairment of Cystatin c-dependent hippocampal neurogenesis by chronic high fa t diet

Piyanart Jiranugrom[1,2], Ik Dong Yoo[3], Min Woo Park[4], Ji Hwan Ryu[5], Jo ng-Seok Moon[4,*] and Sun Shin Yi[1,*]

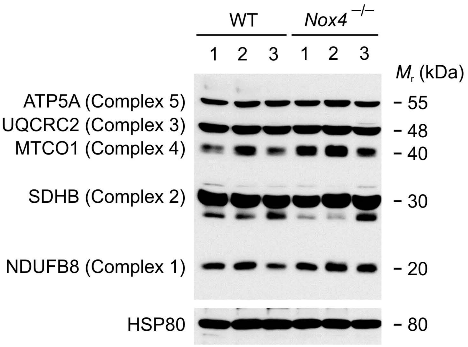

Supplemental Figure S1. The expression of mitochondrial oxidative phosphorylation complex enzymes in fat tissues was comparable between *Nox4*$^{-/-}$ and WT mice. Representative immunoblot analysis for ATP5A (Complex 5), UQCRC2 (Complex 3), MTCO1 (Complex 4), SDHB (Complex 2) and NDUFB8 (Complex 1) of fat tissues from *Nox4*$^{-/-}$ and WT mice (n = 3 per group). HSP80 is used as loading control. ATP5A = ATP synthase F1 subunit alpha; UQCRC2 = ubiquinol-cytochrome c reductase core protein 2; MTCO1 = Mitochondrially Encoded Cytochrome C Oxidase 1; SDHB = Succinate Dehydrogenase Complex Iron Sulfur Subunit B; NDUFB8 = NADH:Ubiquinone Oxidoreductase Subunit B8

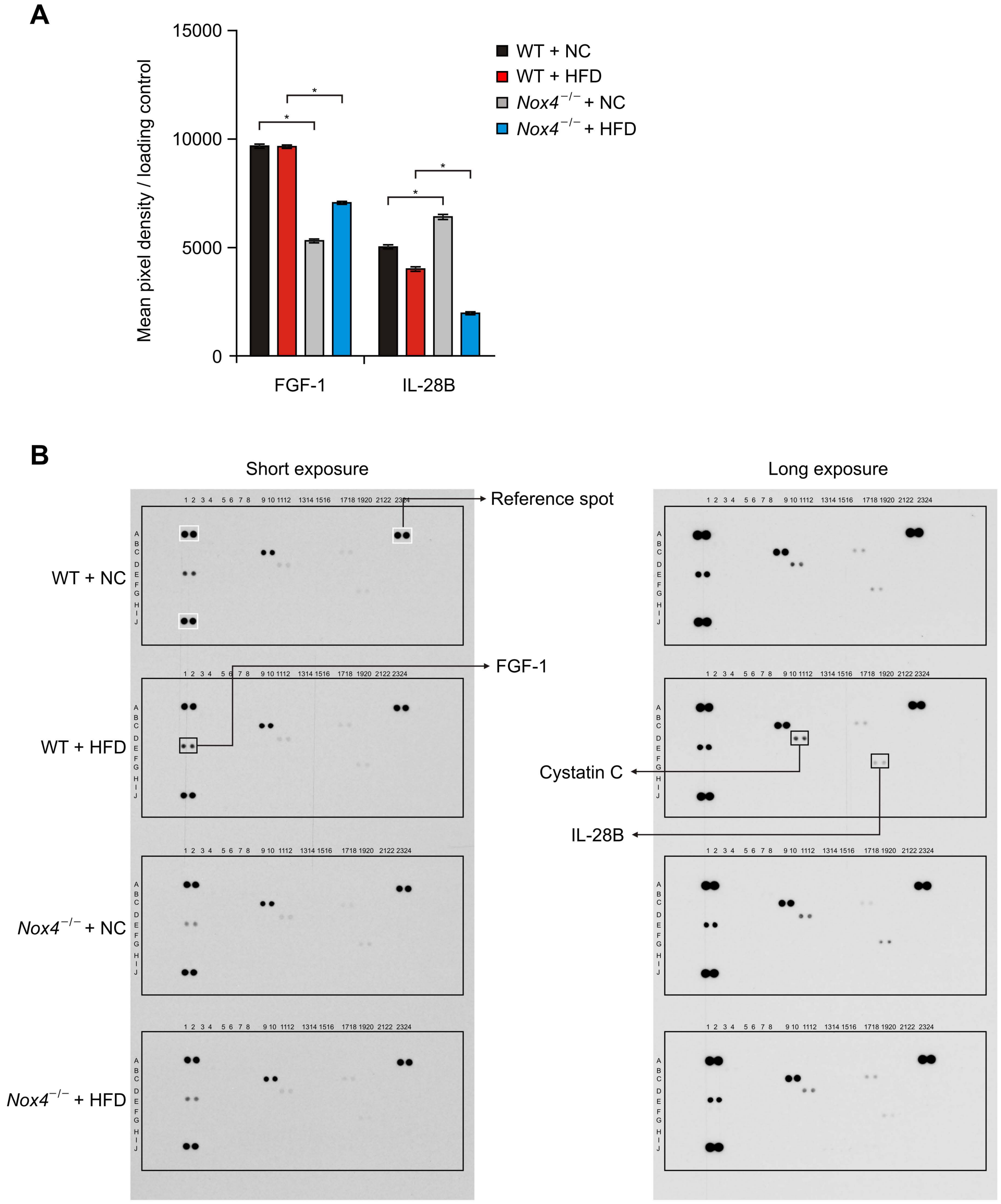

Supplemental Figure S2. The levels of Cystatin C, FGF-1 and IL-28B in hippocampus from *Nox4*$^{-/-}$ and WT mice.

(A) Quantification of FGF-1 and IL-28B secretion from *Nox4* $^{-/-}$ and WT mice with normal chow diet (NC) or high fat diet (HFD). (B) Representative immunoblot analysis for Cystatin C, FGF-1, and IL-28B of hippocampus from *Nox4* $^{-/-}$ and WT mice with normal chow diet (NC) or high fat diet (HFD) (black box). Reference spots are used as loading control (white box). Data are representative of two independent experiments. The 111 different cytokines, chemokines, growth factors or extracellular signaling molecules were analyzed by immunoblot analysis. The location of analytes is marked by A1 to J24 using X axis (1 to 24) and Y (A to J) axis. The values of mean pixel density for whole analytes are described in supplemental Table 1. Data mean ± SD. **P* < 0.05 by Student's two-tail

Supplemental Table 1. Mean pixel density of analytes

Coordinate	Analyte	Entrez Gene ID	Mean pixel density			
			WT + NC	WT + HFD	*Nox4*$^{-/-}$ + NC	*Nox4*$^{-/-}$ + HFD
A1, A2	Reference Spots	N/A	75241 ± 152	76053 ± 207	75349 ± 185	76321 ± 142
A3, A4	Adiponectin/Acrp30	11450	ND	ND	ND	ND
A5, A6	Amphiregulin	11839	ND	ND	ND	ND
A7, A8	Angiopoietin-1	11600	ND	ND	ND	ND
A9, A10	Angiopoietin-2	11601	ND	ND	ND	ND
A11, A12	Angiopoietin-like 3	30924	ND	ND	ND	ND
A13, A14	BAFF/BLyS/TNFSF13B	24099	ND	ND	ND	ND
A15, A16	C1q R1/CD93	17064	ND	ND	ND	ND
A17, A18	CCL2/JE/MCP-1	20296	ND	ND	ND	ND
A19, A20	CCL3/CCL4/MIP-1α/β	20302/20303	ND	ND	ND	ND
A21, A22	CCL5/RANTES	20304	ND	ND	ND	ND
A23, A24	Reference Spots	N/A	78751 ± 244	79125 ± 181	78912 ± 231	79934 ± 227
B3, B4	CCL6/C10	20305	ND	ND	ND	ND
B5, B6	CCL11/Eotaxin	20292	ND	ND	ND	ND
B7, B8	CCL12/MCP-5	20293	ND	ND	ND	ND
B9, B10	CCL17/TARC	20295	ND	ND	ND	ND
B11, B12	CCL19/MIP-3β	24047	ND	ND	ND	ND
B13, B14	CCL20/MIP-3α	20297	ND	ND	ND	ND
B15, B16	CCL21/6Ckine	18829	ND	ND	ND	ND
B17, B18	CCL22/MDC	20299	ND	ND	ND	ND
B21, B22	CD40/TNFRSF5	21939	ND	ND	ND	ND
C3, C4	CD160	54215	ND	ND	ND	ND
C5, C6	Chemerin	71660	ND	ND	ND	ND
C9, C10	Coagulation Factor III/Tissue Factor	14066	26521 ± 125	26754 ± 101	26635 ± 135	26854 ± 147
C11, C12	Complement Component C5/C5a	15139	ND	ND	ND	ND
C13, C14	Complement Factor D	11537	ND	ND	ND	ND
C15, C16	C-Reactive Protein/CRP	12944	ND	ND	ND	ND
C17, C18	CX3CL1/Fractalkine	20312	9851 ± 101	9785 ± 115	9817 ± 112	9874 ± 109
C19, C20	CXCL1/KC	14825	ND	ND	ND	ND
C21, C22	CXCL2/MIP-2	20310	ND	ND	ND	ND
D1, D2	CXCL9/MIG	17329	ND	ND	ND	ND
D3, D4	CXCL10/IP-10	15945	ND	ND	ND	ND
D5, D6	CXCL11/I-TAC	56066	ND	ND	ND	ND
D7, D8	CXCL13/BLC/BCA-1	55985	ND	ND	ND	ND
D9, D10	CXCL16	66102	ND	ND	ND	ND
D11, D12	Cystatin C	13010	48524 ± 235	49721 ± 168	41236 ± 195	25236 ± 157
D13, D14	DKK-1	13380	ND	ND	ND	ND
D15, D16	DPPIV/CD26	13482	ND	ND	ND	ND
D17, D18	EGF	13645	ND	ND	ND	ND
D19, D20	Endoglin/CD105	13805	ND	ND	ND	ND
D21, D22	Endostatin	12822	ND	ND	ND	ND
D23, D24	Fetuin A/AHSG	11625	ND	ND	ND	ND
E1, E2	FGF acidic (FGF-1)	14164	9875 ± 117	9851 ± 138	5104 ± 172	7632 ± 152
E3, E4	FGF-21	56636	ND	ND	ND	ND
E5, E6	Flt-3 Ligand	14256	ND	ND	ND	ND
E7, E8	Gas 6	14456	ND	ND	ND	ND
E9, E10	G-CSF	12985	ND	ND	ND	ND

E11, E12	GDF-15	23886	ND	ND	ND	ND
E13, E14	GM-CSF	12981	ND	ND	ND	ND
E15, E16	HGF	15234	ND	ND	ND	ND
E17, E18	ICAM-1/CD54	15894	ND	ND	ND	ND
E19, E20	IFN-γ	15978	ND	ND	ND	ND
E21, E22	IGFBP-1	16006	ND	ND	ND	ND
E23, E24	IGFBP-2	16008	ND	ND	ND	ND
F1, F2	IGFBP-3	16009	ND	ND	ND	ND
F3, F4	IGFBP-5	16011	ND	ND	ND	ND
F5, F6	IGFBP-6	16012	ND	ND	ND	ND
F7, F8	IL-1α/IL-1F1	16175	ND	ND	ND	ND
F9, F10	IL-1β/IL-1F2	16176	ND	ND	ND	ND
F11, F12	IL-1ra/IL-1F3	16181	ND	ND	ND	ND
F13, F14	IL-2	16183	ND	ND	ND	ND
F15, F16	IL-3	16187	ND	ND	ND	ND
F17, F18	IL-4	16189	ND	ND	ND	ND
F19, F20	IL-5	16191	ND	ND	ND	ND
F21, F22	IL-6	16193	ND	ND	ND	ND
F23, F24	IL-7	16196	ND	ND	ND	ND
G1, G2	IL-10	16153	ND	ND	ND	ND
G3, G4	IL-11	16156	ND	ND	ND	ND
G5, G6	IL-12 p40	16160	ND	ND	ND	ND
G7, G8	IL-13	16163	ND	ND	ND	ND
G9, G10	IL-15	16168	ND	ND	ND	ND
G11, G12	IL-17A	16171	ND	ND	ND	ND
G13, G14	IL-22	50929	ND	ND	ND	ND
G15, G16	IL-23	83430	ND	ND	ND	ND
G17, G18	IL-27 p28	246779	ND	ND	ND	ND
G19, G20	IL-28A/B	330496/338374	4925 ± 235	4147 ± 258	6874 ± 264	2152 ± 154
G21, G22	IL-33	77125	ND	ND	ND	ND
G23, G24	LDL R	16835	ND	ND	ND	ND
H1, H2	Leptin	16846	ND	ND	ND	ND
H3, H4	LIF	16878	ND	ND	ND	ND
H5, H6	Lipocalin-2/NGAL	16819	ND	ND	ND	ND
H7, H8	LIX	20311	ND	ND	ND	ND
H9, H10	M-CSF	12977	ND	ND	ND	ND
H11, H12	MMP-2	17390	ND	ND	ND	ND
H13, H14	MMP-3	17392	ND	ND	ND	ND
H15, H16	MMP-9	17395	ND	ND	ND	ND
H17, H18	Myeloperoxidase	17523	ND	ND	ND	ND
H19, H20	Osteopontin (OPN)	20750	ND	ND	ND	ND
H21, H22	Osteoprotegerin/TNFRSF11B	18383	ND	ND	ND	ND
H23, H24	PD-ECGF/Thymidine phosphorylase	72962	ND	ND	ND	ND
I1, I2	PDGF-BB	18591	ND	ND	ND	ND
I3, I4	Pentraxin 2/SAP	20219	ND	ND	ND	ND
I5, I6	Pentraxin 3/TSG-14	19288	ND	ND	ND	ND
I7, I8	Periostin/OSF-2	50706	ND	ND	ND	ND
I9, I10	Pref-1/DLK-1/FA1	13386	ND	ND	ND	ND
I11, I12	Proliferin	18811	ND	ND	ND	ND
I13, I14	Proprotein Convertase 9/PCSK9	100102	ND	ND	ND	ND
I15, I16	RAGE	11596	ND	ND	ND	ND

I17, I18	RBP4	19662	ND	ND	ND	ND
I19, I20	Reg3G	19695	ND	ND	ND	ND
I21, I22	Resistin	57264	ND	ND	ND	ND
J1, J2	Reference Spots	N/A	71085 ± 107	70936 ± 174	71524 ± 121	71852 ± 184
J3, J4	E-Selectin/CD62E	20339	ND	ND	ND	ND
J5, J6	P-Selectin/CD62P	20344	ND	ND	ND	ND
J7, J8	Serpin E1/PAI-1	18787	ND	ND	ND	ND
J9, J10	Serpin F1/PEDF	20317	ND	ND	ND	ND
J11, J12	Thrombopoietin	21832	ND	ND	ND	ND
J13, J14	TIM-1/KIM-1/HAVCR	171283	ND	ND	ND	ND
J15, J16	TNF-α	21926	ND	ND	ND	ND
J17, J18	VCAM-1/CD106	22329	ND	ND	ND	ND
J19, J20	VEGF	22339	ND	ND	ND	ND
J21, J22	WISP-1/CCN4	22402	ND	ND	ND	ND
J23, J24	Negative Control	N/A	ND	ND	ND	ND

* ND : Not detectable
